# sST2 as a New Biomarker of Chronic Kidney Disease-Induced Cardiac Remodeling: Impact on Risk Prediction

**DOI:** 10.1155/2018/3952526

**Published:** 2018-10-08

**Authors:** Maëlle Plawecki, Marion Morena, Nils Kuster, Leila Chenine, Hélène Leray-Moragues, Bernard Jover, Pierre Fesler, Manuela Lotierzo, Anne-Marie Dupuy, Kada Klouche, Jean-Paul Cristol

**Affiliations:** ^1^Département de Biochimie-Hormonologie, CHU Montpellier, Université de Montpellier, Montpellier, France; ^2^PhyMedExp, Université de Montpellier, CNRS, INSERM, Montpellier, France; ^3^Département de Néphrologie, CHU Montpellier, Université de Montpellier, Montpellier, France; ^4^Département de Médecine Interne et Hypertension, CHU Montpellier, Université de Montpellier, Montpellier, France; ^5^Département de Réanimation, CHU Montpellier, Université de Montpellier, Montpellier, France

## Abstract

Heart failure is the most frequent cardiac complication of chronic kidney disease (CKD). Biomarkers help identify high-risk patients. Natriuretic peptides (BNP and NT-proBNP) are largely used for monitoring patients with cardiac failure but are highly dependent on glomerular filtration rate (GFR). Soluble suppression of tumorigenicity 2 (sST2) biomarker is well identified in risk stratification of cardiovascular (CV) events in heart failure. Furthermore, sST2 is included in a bioclinical score to stratify mortality risk. The aims of this study were to evaluate (i) the interest of circulating sST2 level in heart dysfunction and (ii) the bioclinical score (Barcelona Bio-Heart Failure risk calculator) to predict the risk of composite outcome (major adverse coronary events) and mortality in the CKD population. A retrospective study was carried out on 218 CKD patients enrolled from 2004 to 2015 at Montpellier University Hospital. sST2 was measured by ELISA (Presage ST2® kit). GFR was estimated by the CKD-EPI equation (eGFR). Indices of cardiac parameters were performed by cardiac echography. No patient had reduced ejection fraction. 112 patients had left ventricular hypertrophy, and 184 presented cardiac dysfunction, with structural, functional abnormalities or both. sST2 was independent of age and eGFR (*ρ* = 0.05, *p* = 0.44, and *ρ* = −0.07, *p* = 0.3, respectively). Regarding echocardiogram data, sST2 was correlated with left ventricular mass index (*ρ* = 0.16, *p* = 0.02), left atrial diameter (*ρ* = 0.14, *p* = 0.04), and volume index (*ρ* = 0.13, *p* = 0.05). sST2 alone did not change risk prediction of death and/or CV events compared to natriuretic peptides. Included in the Barcelona Bio-Heart Failure (BCN Bio-HF) score, sST2 added value and better stratified the risk of CV events and/or death in CKD patients (*p* < 0.0001). To conclude, sST2 was associated with cardiac remodeling independently of eGFR, unlike other cardiac biomarkers. Added to the BCN Bio-HF score, the risk stratification of death and/or CV events in nondialyzed CKD patients was highly improved.

## 1. Introduction

CV events and death are associated with reduced eGFR [1]. The prevalence of most comorbid conditions, including heart failure (HF), increases with decreasing eGFR [2]. Heart failure with preserved ejection fraction (HFpEF) constitutes the main feature of uremic cardiopathy and is often referred to as type 4 cardiorenal syndrome [3, 4]. In May 2016, the European Society of Cardiology developed guidelines to help diagnosis of chronic HFpEF, including cardiac structural or functional alterations underlying HF [5]. Left ventricular hypertrophy (LVH) represents the major event in type 4 cardiorenal syndrome (chronic renocardiac damage). The prevalence of LVH is estimated between 16% and 31% in CKD patients with eGFR > 30 mL/min to reach 60 to 75% before dialysis and 90% after dialysis [6]. Diastolic dysfunction, defined by pseudonormal or restrictive pattern through tissue Doppler imaging (E/e' ≥ 10) which appears in the early stages of CKD, now emerges as an independent predictor of mortality and development of HF in a CKD patient [7].

Recent studies have identified new biomarkers involved in the pathogenesis of remodeling and cardiac fibrosis. Among them is sST2, an emerging biomarker predictive of fibrosis and cardiac remodeling in HF patients without CKD. This is a marker of interest in the stratification of patients at risk as well as in the therapeutic response of HF patients [8–11]. ST2 belongs to the family of interleukin receptors of type-1 (IL-1) and exists as membrane-bound (ST2L) and soluble (sST2) isoforms. By binding interleukin-33 (IL-33), ST2L is responsible for antihypertrophic, antifibrotic, and antiapoptotic effects [12]. sST2 is the soluble circulating form which acts as a decoy receptor, sequesters IL-33, and prevents its binding to ST2L, thereby neutralizing the beneficial effects of the ST2L/IL-33 signaling pathway [13]. sST2 is mainly secreted by cardiomyocytes when the cells are subjected to biomechanical overload. Nevertheless, the main source of sST2 secretion is still controversial, and in human cardiac disease, the vascular endothelial cells were shown to be the predominant source of sST2, rather than the human myocardium [14].

In patients with chronic HF episodes, sST2 acts as a predictor of both all-cause and cardiovascular death [15]. sST2 was included in a novel bioclinical algorithm (Barcelona Bio-Heart Failure (BCN Bio-HF) risk calculator) in association with NT-proBNP and high-sensitivity cardiac troponin T (hs-cTnT), which allowed accurate prediction of death at 1, 2, and 3 years in HF patients [16]. High levels of sST2 associated with NT-proBNP and identified risk factors improve prognosis performance independently of left ventricular ejection fraction and renal function in HF [17]. In this context, sST2 measurement can identify patients with left ventricular remodeling and decompensated hemodynamic profile [18].

To our knowledge, only few data are available regarding the prognosis value of fibrosis and myocardial remodeling biomarker in CKD patients for which the risk of a CV event constitutes the main cause of mortality. Therefore, objectives of this study were to evaluate sST2 in cardiac remodeling and to assess its role alone or in combination with other common biological parameters of HF for risk stratification of CV events or/and mortality in a nondialyzed CKD population (BCN Bio-HF score).

## 2. Population and Methods

### 2.1. Patients and Study Design

218 patients were enrolled in the Montpellier University Hospital between 2004 and 2015. Main inclusion criteria were the ability to give informed consent, age > 18 years, cardiac echography at inclusion, and a confirmed diagnosis of CKD according to the National Kidney Foundation and KDIGO Guidelines [19, 20]. Stages of CKD were determined using eGFR calculated using the CKD-EPI equation [21]. None of the patients in stage 5 were on hemodialysis or on peritoneal dialysis. Regarding antihypertensive treatment, 16 patients had no treatment, 39 were on mono-, 71 on bi-, 70 on tri-, and 22 on quadritherapy.

At the time of enrollment, all patients had an echocardiogram performed by a trained physician. Dry and heparinized blood samples were drawn, and serum/plasma stored at −80°C for further analyses.

The follow-up of all included patients was approximatively 3 years with time-to-event analysis until the occurrence of fatal or nonfatal CV events, defined as major adverse coronary events (MACE).

Written informed consent was obtained for all patients. The protocol was approved by local authorities (Ethics Committee of Montpellier) according to standards currently applied in France (Commission Nationale de l'Informatique et des Libertés, CNIL, N°MR001). A biological collection was also registered by the French government (research ministry, # DC 2008-417 and # DC 2013-2027). The study was done in accordance with the Declaration of Helsinki and Good Clinical Practice guidelines.

### 2.2. Cardiac Echography

Cardiac echocardiography was performed by a trained physician at inclusion. No patients presented signs of heart failure at inclusion. Subclinical cardiac dysfunction was defined as left ventricular ejection fraction (LVEF) > 40% with structural abnormality (left ventricular mass index (LVMI) ≥ 115 g/m^2^ for men and ≥95 g/m^2^ for women or left atrial volume index (LAVI) > 34 mL/m^2^) or functional abnormality with impaired relaxation (E/A < 1) [5].

### 2.3. Laboratory Analyses

Biochemical parameters, including classical cardiac variables (NT-proBNP and high-sensitivity troponin T (hs-cTnT)), were performed on a Cobas 8000/e602 immunochemistry system (Roche Diagnostics, Meylan, France). C-reactive protein (CRP), urea, and IDMS traceable enzymatic creatinine were determined on a Cobas 8000/c701 (Roche Diagnostics, Meylan, France). Intact aminoterminal propeptide of type I procollagen (PINP) as a biomarker of collagen synthesis was determined by chemiluminescence technology using the IDS-iSYS Multi-Discipline automated analyser (IDS, Boldon, England).

### 2.4. sST2 Measurement

sST2 was measured using a sandwich ELISA kit (Presage© ST2 assay, Critical Diagnostics, San Diego, California, distributed in France by Eurobio Laboratories). In chronic HF patients, the upper reference limit for sST2 was 35 ng/mL [22]. A recombinant human sST2 standard calibrator was provided for this assay. sST2 concentrations were measured according to sST2 assay procedures and adapted on Evolis (France). Briefly, 100 *μ*l of standard, diluted samples (1 : 20 in sample diluent) was added to the well of a ready-to-use microtiter plate coated with mouse monoclonal anti-human sST2 antibody. The standard curve was in the concentration range 2.8–100 ng/mL. Then, the plate was incubated for 60 min at room temperature. After washing, 100 *μ*l of biotinylated antibody reagent was added into each well and incubated for 60 min at room temperature. After washing, 100 *μ*l of streptavidin-HRP conjugated was added into each well and incubated for 30 min at room temperature. After washing, the TMB substrate was added to each well and incubated for 20 min at room temperature in the dark. Then, stop solution was added and absorbance was read at 450 nm.

### 2.5. Barcelona Bio-Heart Failure Score

The Barcelona Bio-Heart Failure risk calculator (BCN Bio-HF calculator) estimates the risk of death in patients with HF described by Lupón et al. [16]. The BCN Bio-HF calculator is an algorithm based on eight independent models, depending on available data. It is derived from a real-life cohort and includes, in addition to classical prediction factors, serum NT-proBNP, hs-cTnT, and sST2 reflecting different pathophysiological pathways. The models account for clinical and biological characteristics and treatments to predict the risk of mortality at 1, 2, and 3 years. Pharmacological treatments include beta-blockers, ARBs/ACEI, statins, and furosemide. In our study, all clinical and biomarker variable models were taken into account (hs-cTnT, NT-proBNP, and sST2). Using this model, prognostic indices were computed for each patient in our population.

### 2.6. Statistical Analysis

Descriptive statistics are presented as numbers (percentages) for categorical data and as medians (interquartile range (IQR)) for continuous variables. *χ*^2^ test was performed to investigate the presence of differences between proportions. The Mann-Whitney *U* test and Kruskal-Wallis test were used to compare groups, as appropriate. Since their distributions were skewed, logarithmic transformations of sST2, troponin, NT-proBNP, and PINP biomarkers were used. For correlation analyses, Spearman's rank correlation coefficients were computed. Composite outcome was defined as any of the following events (MACE) during follow-up: death, myocardial infarction, ischemic cardiomyopathy, angioplasty, valvular cardiomyopathy, stroke, vascular angioplasty, or cardiac arrhythmias. The Kaplan-Meier estimator of event-free survival was used to assess the ability of biomarkers to predict adverse outcome in the population. Potential predictors of composite outcome were further evaluated using the Cox proportional hazard regression. The net reclassification improvement (NRI) was used to assess the incremental value by adding a biomarker over the BCN Bio-HF score. No treatment adjustment in relation to cardiac biomarkers and kidney dysfunction was assessed in the BCN Bio-HF score analysis since they were already included in the initial score calculation.

## 3. Results

### 3.1. Population Characteristics

This study included a total of 218 patients at different stages of CKD (i.e., 36 patients at stages 1–2, 42 patients at stage 3A, 57 patients at stage 3B, 62 patients at stage 4, and 21 patients at stage 5). Median eGFR level was 37 mL/min/1.73 m^2^ (IQR 23–52). During the follow-up period, 85 out of 218 patients presented composite outcome. Demographic data, laboratory findings, and echocardiogram parameters in the global population presenting or not presenting composite outcome are shown in Table 1. Among the population, the median LVEF was 62%, and none of the patients had reduced LVEF. 112 (51%) patients presented LVH and 184 (84%) a cardiac dysfunction. Cardiac dysfunction was as follows: 45 patients with structure abnormality only, 72 with function abnormality only, and 67 with both. Median follow-up was 3.0 years (IQR 1.3–6.4) after initial evaluation. Compared to patients free of major adverse coronary events (MACE), eGFR and LVEF were lower in patients with MACE, whereas age, LAVI, left atrial diameter (LAD), LVMI, levels of CRP, NT-proBNP, and hs-cTnT were higher. No significant difference in PINP levels was observed between groups. Median sST2 was 29.5 ng/mL (IQR 22.6–35.1), and 55 out of 218 patients (25%) had an elevated level of sST2 (upper reference limit is 35 ng/mL in chronic HF).

### 3.2. sST2 Is Associated with Cardiac Remodeling Feature

Correlation between sST2 and inflammatory and cardiac biomarkers (CRP, NT-proBNP, and hs-cTnT) was significant (*ρ* = 0.17, *p* = 0.01; *ρ* = 0.14, *p* = 0.03; and *ρ* = 0.15, *p* = 0.03, respectively).

**Figure 1:**
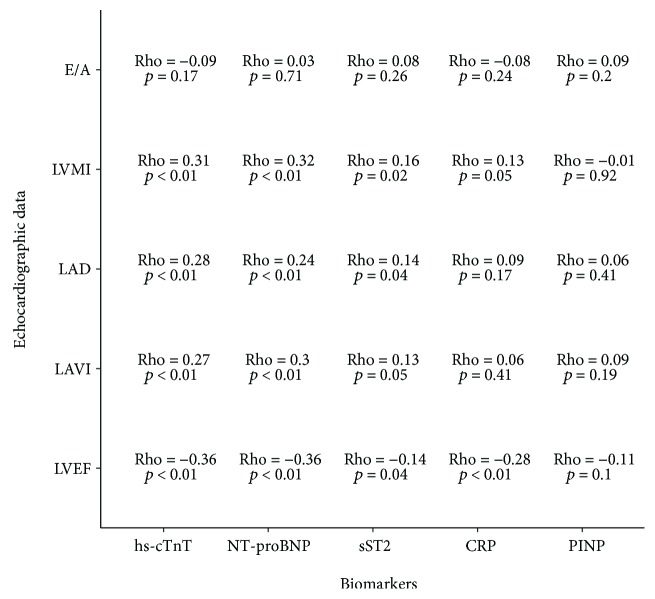
Correlation analysis between echocardiogram data, variables, and biomarkers.

Regarding echocardiogram data, sST2 was correlated with LVEF (*ρ* = −0.14, *p* = 0.04) and cardiac remodeling (i.e., LAD (*ρ* = 0.14, *p* = 0.04), LAVI (*ρ* = 0.13, *p* = 0.05), and LVMI (*ρ* = 0.16, *p* = 0.02)). No association with functional abnormality was observed (E/A (*ρ* = 0.08, *p* = 0.26)). PINP was not correlated with any echocardiographic data (Figure 1). Other cardiac biomarkers (hs-cTnt and NT-proBNP) were correlated with structural abnormality parameters. Concerning patient treatments, no correlation was observed between sST2 and beta-blockers, ARBs/ACEI, statins, or furosemide (data not shown).

### 3.3. sST2 Is Independent of GFR and Age

**Figure 2:**
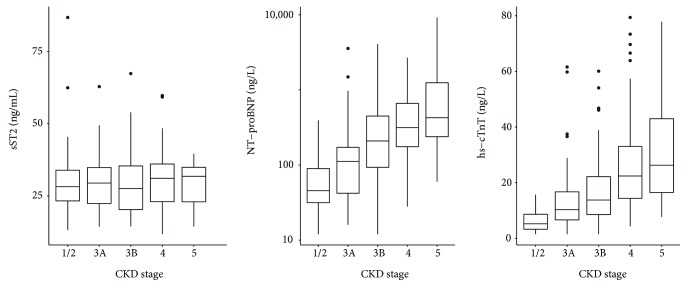
sST2, NT-proBNP, and hs-cTnT levels according to CKD stages (Kruskal-Wallis test: *p* = 0.9, *p* < 0.001, and *p* < 0.001, respectively).

No correlation between sST2 and both age (*ρ* = 0.05, *p* = 0.44) or eGFR (*ρ* = −0.07, *p* = 0.3) was observed, in contrast to classical cardiac biomarkers such as hs-cTnT (*ρ* = 0.55, *p* < 0.001, and *ρ* = −0.59, *p* < 0.001, respectively) and NT-proBNP (*ρ* = 0.51, *p* < 0.001, and *ρ* = −0.56, *p* < 0.001, respectively). Moreover, no relationship was observed between sST2 and CKD stages (*p* = 0.9) whereas NT-proBNP and hs-cTnT values increased from stages 1 to 5 (*p* < 0.001 for both parameters) (Figure 2).

### 3.4. Predictors of Outcome

During the 3 years of median follow-up (IQR 1.3–6.4), 85 (39%) patients experienced the composite outcome of death and/or CV events.

In univariate Cox analysis, older age (HR 1.052 (1.031–1.072)), male gender (female HR 0.420 (0.251–0.704)), increased LAVI (HR 1.050 (1.015–1.086)) and LAD (HR 1.074 (1.030–1.119)), elevated hs-cTnT (HR 5.152 (2.659–9.983)), NT-proBNP (HR = 1.650 (1.116–2.439)), and CRP (HR 2.155 (1.388–3.346)) were related to composite outcome (Table 2).

### 3.5. Multimarker Strategy Based on Barcelona Bio-HF Score

**Figure 3:**
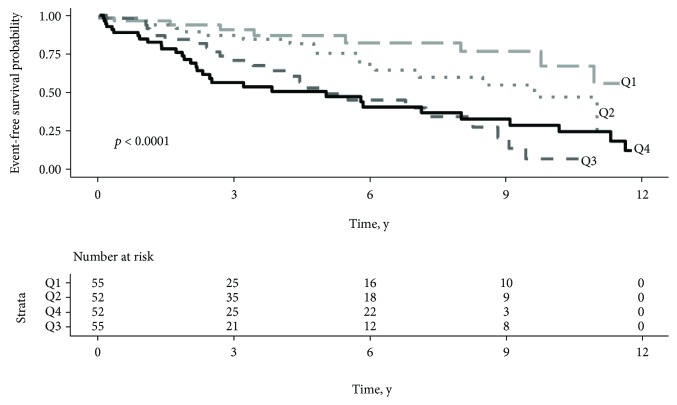
Barcelona Bio-HF score and composite outcome prediction. Event-free survival probability according to mortality risk predicted by the Barcelona Bio-Heart Failure score. Study population was stratified by quartiles of 1-year mortality risk (Q1: risk < 1.56%, Q2: 1.56 ≤ risk < 3.45%, Q3: 3.45 ≤ risk < 6.68%, and Q4: risk > 6.68%). *p* value refers to the log-rank test.

The Barcelona Bio-HF score was applied to assess the predictive composite outcome in our cohort. Taken together, NT-proBNP, hs-cTnT, and sST2 were highly predictive of the composite outcome of cardiovascular events and/or death (*p* < 0.0001) (Figure 3). A combination of CRP with the Barcelona Bio-HF score was performed in order to identify a high-risk subgroup and to improve the risk of CV events or death composite outcome. The risk classification analysis including the CRP level did not allow better patient classification (continuous NRI = 16% (−11.7–32.8%), *p* = 0.27).

## 4. Discussion

This study shows that the sST2 level is associated with cardiac remodeling features, and unlike common cardiac biomarkers, this biomarker is independent of eGFR and age. A multimarker approach including sST2 is thus reported as an appealing tool in CV risk stratification of nondialyzed CKD patients.

### 4.1. sST2 and Heart Dysfunction in CKD Patients

HFpEF is associated with increased cardiac remodeling, abnormal cardiac mechanics, and poor outcomes in CKD patients [1, 23]. Brain natriuretic peptides and hs-cTnT can facilitate the diagnosis of HF among patients with CKD. Elevation of these biomarkers is related to cardiac modifications contributing to HF [24]. Vickery et al. reported that eGFR and cardiac dysfunction have independent effects on brain natriuretic peptide concentrations in CKD patients [25]. A reduced renal excretion provokes elevated levels of cardiac troponins, NT-proBNP, and in a lesser extent BNP limiting the utility of these markers although higher brain natriuretic peptide levels remain predictive of increased mortality in CKD [26–28]. sST2 is an interesting biomarker, and most studies have described sST2 as an independent marker of renal function and hemodialysis [29–31]. We confirmed here that sST2 is not correlated with eGFR, unlike NT-proBNP, and no difference was observed among CKD stages. Therefore, the weak correlation observed between NT-proBNP and sST2 (*ρ* = 0.14, *p* = 0.03) can be explained by renal dysfunction as a confusing factor (Figure 2). In a study conducted by Bao et al., sST2 levels were higher in CKD patients compared to healthy controls and were correlated with disease severity [32]. More recently, Gungor et al. observed that sST2 levels increased with CKD stages [33]. Indeed, these investigations did not evaluate cardiac function, and sST2 elevation was reported to be involved in the inflammatory state. Moreover, sST2 measurements are not directly comparable because ELISA kits with different standards or antibodies were used. Lastly, in both studies, GFR was estimated through the Modification of Diet in Renal Disease Equation, and this could lead to different GFR values depending on the estimation method used.

sST2 is involved in pathophysiology of cardiac fibrosis, and its increase is considered as an indirect circulating marker of cumulative fibrotic processes [34]. Regarding echocardiogram results, sST2 correlates significantly with variables that describe structural alterations, and no correlation with functional abnormalities was observed. We can thus speculate that in CKD patients, sST2 by decreasing the availability of IL-33 may be involved in cardiac remodeling features typically observed in HFpEF. Although other cardiac biomarkers showed better correlation with cardiac dysfunction, sST2 is quite relevant because it does not depend on age and renal function. These results represent an important step in early detection of cardiac performance alteration in CKD. Finally, no correlation between PINP and echocardiographic data was observed. This could be explained by the lack of specificity of this marker in cardiac fibrosis [35].

### 4.2. sST2 and Prognosis Value

To our knowledge, this is the first time we observe that a multimarker strategy including combined sST2, NT-proBNP, and hs-cTnT biomarkers is highly associated with cardiovascular events and/or mortality and suitable in nondialyzed CKD patient risk stratification.

sST2 represents a promising biomarker in prognosis mortality and CV events in chronic HF [36, 37]. Recently, an update of ACC/AHA guidelines stated that the use of myocardial fibrosis biomarkers such as sST2 might be considered for predicting risk of hospitalization and death in patients with chronic HF and potentially added to natriuretic peptide biomarker levels in their prognostic value [34]. Our study confirms that hs-cTnT, CRP, and to a lesser extent NT-proBNP alone are predictive of poor outcome, as described in a hemodialysis population [38]. Yet, sST2 alone does not allow CV events or death composite outcome prognosis in nondialyzed CKD patients. Our findings are in line with Keddis et al., who found that sST2 level did not change CV risk prediction compared to cardiac troponin T in patients considered for kidney transplant [39].

A multimarker strategy approach was developed and proven to be more informative than a single biomarker in HF prognosis. To date, only few clinical scores evaluating risk stratification of HF have been developed. The BCN Bio-HF score is a unique tool combining a panel of biomarkers and clinical variables [40]. Taken together, biomarkers provide information about myocyte necrosis (hs-cTnT), fibrosis and inflammation (sST2), and chamber strain (NT-proBNP). In our study, NT-proBNP, hs-cTnT, and sST2 in combination with clinical variables and treatments were highly predictive of composite outcome. We showed that the BCN Bio-HF score can also be applied to the CKD population and is highly predictive of CV events and mortality. Beside, adding CRP to the BCN Bio-HF score did not provide better reclassification of CKD patients, as observed for HF [41]. Although CRP is considered as a marker of early inflammation and a low level of hs-CRP may be associated with a more favorable prognosis in patients with coronary heart disease [42], its level remains stable in CKD before dialysis and unlikely plays a major role in subacute inflammation [43]. Our results suggest that CRP does not improve the prognosis of patients at nondialyzed CKD stages, presenting mainly a subclinical cardiac dysfunction. In this population, a multimarker strategy such as the BCN Bio-HF score is definitely helpful to better stratify risk of death and CV events. Further longitudinal studies in CKD patients are needed to better characterize the interest of these combined markers involved in HF pathophysiology.

## 5. Limitations

This study presents several limitations. First of all, tissue Doppler imaging was not taken into account at the time of inclusion; consequently, the e' wave was not available. Then, a relatively small cohort of patients from a single medical center was enrolled. We only measured biomarkers at time of recruitment and did not evaluate the long-term trends of biomarkers, which could be useful for patient follow-up. Moreover, sST2 assay is not available in all laboratories, which reduces the use of the multimarker strategy.

## 6. Conclusion

To our knowledge, this is the first study showing that sST2 is a good biomarker to evaluate the cardiac remodeling feature in nondialyzed CKD patients. Although sST2 alone is not predictive of CV events and death in this population, it plays an important role to stratify risk of all-cause mortality and CV events in a multimarker strategy combined with clinical variables.

## Figures and Tables

**Table 1:** Baseline characteristics of all patients, with and without MACE.

Variable	Study population (*n* = 218)	No MACE (*n* = 133)	MACE (*n* = 85)	*p*
Age (years)	68.31 [57.62–75.47]	63.35 [49.18–71.69]	71.75 [67.44–79.37]	<0.001
Gender				0.005
Male	139 (64%)	75 (56%)	64 (75%)	
Female	79 (36%)	58 (44%)	21 (25%)	
Follow-up (years)	3.0 [1.3–6.4]	3.0 [1.3–6.5]	2.8 [1.4-5.8]	0.869
eGFR (mL/min/1.73 m^2^)	37 [23–52]	40 [26–57]	35 [22–44]	0.014
SBP (mmHg)	134 [120–146]	135 [122–146]	130 [120–146]	0.214
DBP (mmHg)	73 [69–80]	75 [70–80]	70 [65–80]	0.051
*Echocardiography*				
LVEF (%)	62 [58–65]	65 [60–67]	60 [54–64]	<0.001
E/A	0.85 [0.70–1.11]	0.87 [0.73–1.12]	0.8 [0.69–1.11]	0.204
LAVI (mL/m^2^)	11.4 [7.6–14.9]	10.4 [7.0–14.1]	13.3 [9.1–16.3]	0.001
LAD (mm)	34 [30–37]	33 [29–36]	36 [32–39]	<0.001
LVMI (g/m^2^)	109.9 [85.3–130.5]	106.8 [79.0–125.0]	118.0 [97.2–139.3]	0.004
*Biomarkers*				
NT-proBNP (ng/L)	182.5 [75.0–445.3]	129.0 [61.0–379.0]	287.0 [121.2–623.5]	<0.001
sST2 (ng/mL)	29.5 [22.6–35.1]	28.2 [21.7–34.3]	30.5 [24.3–36.7]	0.100
PINP (ng/mL)	52.2 [38.2–77.5]	51.95 [38.2–77.2]	55.3 [38.4–77.5]	0.522
hs-cTnT (ng/L)	14.3 [7.7–24.9]	11.7 [6.2–19.5]	19.1 [12.4–34.3]	<0.001
CRP (mg/L)	2.2 [1.1–4.7]	1.8 [0.8–3.6]	3.2 [1.6–6.9]	<0.001
Na (mmol/L)	141 [139–142]	141 [139–142]	140 [139–142]	0.829
Hb (g/dL)	13.2 [12.3–14.2]	13.4 [12.2–14.4]	13.0 [12.4–14.0]	0.702
*Treatments*				
Beta-blockers				0.054
No	118 (54.1%)	79 (59.4%)	39 (45.9%)	
Yes	100 (45.9%)	54 (40.6%)	46 (54.1%)	
ARBs/ACEI				0.274
No	55 (25.2%)	30 (22.6%)	25 (29.4%)	
Yes	163 (74.8%)	103 (77.4%)	60 (70.6%)	
Statins				0.061
No	120 (55.0%)	80 (60.2%)	40 (47.1%)	
Yes	98 (45.0%)	53 (39.8%)	45 (52.9%)	
Furosemide				0.001
No	100 (45.9%)	73 (54.9%)	27 (31.8%)	
Yes	118 (54.1%)	60 (45.1%)	58 (68.2%)	

Data presented as median [1^st^ quartile–3^rd^ quartile] for quantitative variables and proportions for categorical variables. ACEI: angiotensin converting enzyme inhibitors, ARBs: angiotensin receptor blockers, DBP: diastolic blood pressure, eGFR: estimated glomerular filtration rate, LAD: left atrial diameter, LAVI: left atrial volume index, LVEF: left ventricular ejection fraction, LVMI: left ventricular mass index, MACE: major adverse coronary events, SBP: systolic blood pressure. *p* value was determined by *χ*^2^ and Mann-Whitney *U* tests.

**Table 2:** Univariate Cox analysis predictive of cardiovascular events and/or death composite outcome.

Variable	HR [95% CI]	*p*
Age (years)	1.052 [1.031–1.072]	<0.001
Female	0.420 [0.251–0.704]	0.001
SBP (mmHg)	0.992 [0.981–1.004]	0.209
DBP (mmHg)	0.986 [0.966–1.006]	0.167
LAVI (mL/m^2^)	1.050 [1.015–1.086]	0.005
LAD (mm)	1.074 [1.030–1.119]	0.001
LVMI (g/m^2^)	1.004 [0.998–1.009]	0.183
E/A	1.010 [0.607–1.681]	0.969
Log hs-cTnT (ng/L)	5.152 [2.659–9.983]	<0.001
Log NT-proBNP (ng/L)	1.650 [1.116–2.439]	0.012
Log CRP (mg/L)	2.155 [1.388–3.346]	0.001
Log sST2 (ng/mL)	2.836 [0.532–15.134]	0.222
Log PINP (ng/mL)	0.836 [0.340–2.057]	0.696
eGFR (mL/min/1.73 m^2^)	0.992 [0.981–1.003]	0.172
Na (mmol/L)	0.985 [0.908–1.069]	0.720
Hb (g/dL)	0.991 [0.852–1.153]	0.909

DBP: diastolic blood pressure, eGFR: estimated glomerular filtration rate, Hb: hemoglobin, LAD: left atrial diameter, LAVI: left atrial volume index, LVMI: left ventricular mass index, SBP: systolic blood pressure.

## Data Availability

The datasets generated and/or analysed during the current study are available from the corresponding author on reasonable request.
